# A new high protein-to-energy enteral formula with a whey protein hydrolysate to achieve protein targets in critically ill patients: a prospective observational tolerability study

**DOI:** 10.1038/s41430-021-00956-9

**Published:** 2021-06-24

**Authors:** Franziska Tedeschi-Jockers, Simona Reinhold, Alexa Hollinger, Daniel Tuchscherer, Caroline Kiss, Lukas Gantner, Katrin Ledergerber, Sibylle Zimmermann, Jonas Scheuzger, Jan Huber, Martin Siegemund

**Affiliations:** 1 Intensive Care Unit, University Hospital Basel, Basel, Switzerland; 2 Department of Clinical Nutrition, University Department of Geriatric Medicine Felix Platter, Basel, Switzerland

**Keywords:** Phase IV trials, Ethics

## Abstract

**Objectives:**

Current guidelines and expert recommendations stress the need to implement enteral feeds with a higher protein-to-energy ratio to meet protein requirements as recommended while avoiding gastrointestinal side effects and energy overfeeding in ICU patients.

**Materials and methods:**

Prospective tolerability study in 18 critically ill patients with a high protein formula (high protein-to-energy (HP:E) formula = Fresubin® Intensive; HPG) compared to a contemporary matched conventional therapy group (CTG). The primary outcome was GI intolerance defined as ≥300 ml daily gastric residual volume (GRV), vomiting, or diarrhea on days 1 and 2. Secondary outcomes were the percentage of patients reaching their protein target on day 4 and overall protein intake.

**Results:**

Groups were comparable regarding demographic characteristics, disease severity, organ failures, mechanical ventilation, and NUTRIC score at baseline. Eighteen patients completed the 4-day feeding period. The number of events of GRV of ≥300 ml/day was equal in both groups (33.3%). The incidence of diarrhea and vomiting was low in the HPG (two patients concerned). EN did not need to be discontinued due to intolerance in any group. Seventy-two percent of patients reached protein targets ≥1.3 g/kgBW/d within 4 days after initiation of enteral feeding, which was superior to the CTG (33%). Post-hoc testing showed group differences of protein intake between HPG and CTG were significant at *t* = 72 h and *t* = 96 h. Energy targets were met in both groups.

**Conclusion:**

The HP:E formula containing 33% whey protein hydrolysate is well tolerated in this tolerability study. Due to the HP:E ratio protein targets can be reached faster. Larger randomized trials are needed to confirm preliminary results.

**Trial registration:**

ClinicalTrials.gov Identifier: NCT02678325. Registered 2 May 2016.

## Background

Besides treatment for the underlying disease or insult, providing adequate nutritional support, according to current guidelines, is vital to counteract muscle wasting and loss of functional capacity associated with a critical illness. Due to its reduction of infectious complications, shorter ICU, and hospital stay [1], the enteral route is prioritized over parenteral nutrition (PN) [1–4]. Appropriate timing of nutritional support containing the required amounts of energy and protein in intensive care unit (ICU) patients has received considerable attention, in particular during the acute phase of critical illness [1, 5–12]. Based on the available evidence mainly from large observational studies, current guidelines and expert recommendations support a protein intake in the range of 1.2–2.0 g/kg body weight (BW) per day for critically ill patients [1, 2, 13, 14]. While recommended protein targets for ICU patients have increased over time, energy targets were concurrently reduced, in particular for the acute phase of critical illness, which has been divided into “early period” for day 1 and 2, and “late period”, for days 3–7 [1]. It is now generally accepted that energy targets in critical illness should be defined by indirect calorimetry (IC) whenever possible [1, 2]. If this tool is not available, oxygen consumption (VO_2_) from pulmonary arterial catheter or carbon dioxide production derived from the ventilator (VCO_2_) has been suggested as an alternative by Staples et al. [15]. In absence of the above mentioned IC, VO_2_, and VCO_2_, a maximum of 20–25 kcal/kg BW/day should be provided during the acute phase with a possible increase to 25–30 kcal/kg/day in stable patients during the later phase of their ICU stay [1, 2, 16].

Providing adequate amounts of protein and energy is of utmost importance for critically ill patients who are at high risk of malnutrition. In particular, during the acute phase of critical illness, the breakdown of muscle protein is enhanced to provide amino acids to foster the synthesis of acute-phase proteins, immune function, gluconeogenesis, and wound healing [17, 18]. Inadequate nutrition support and immobility further aggravate muscle wasting and depletion of energy stores, resulting in prolonged morbidity and persistent physical weakness [11, 19]. However, the optimal amount of protein intake in critically ill patients is still a matter of debate [20].

Observational studies point at the possibility that a higher protein intake, especially during the early phase of critical illness, improves outcome and might reduce mortality [21–23]. However, the only study up to date, which examines the correlation between protein intake and reduced mortality using randomized controlled trials (RCT), is a meta-analysis that looks at RCTs between 1966 and 2015 and does not support the latter findings [24]. Yet, the doses of protein delivered in this review did not meet current guidelines, as they were only up to 1.02 g/kg/day. Furthermore, only two out of 14 studies varied the allotment of protein whilst allocating the same amount of energy [2, 16].

The implementation of lower energy targets in line with guidelines to prevent overfeeding carries an associated risk of insufficient protein intake because nutritional prescriptions are usually based on energy targets rather than on protein targets. In fact, protein targets according to guidelines are rarely achieved with conventional enteral formulations providing only 40–60 g of protein per 1000 kcal. The possible resulting protein deficit can be critical in the acute phase of critical illness and has been shown to be associated with prolonged ventilation time and ICU stays in observational studies [6]. Another challenge in the provision of adequate amounts of protein is feeding intolerance, occurring with a prevalence of up to 75% in enterally fed critically ill patients [25]. Hence, ICU patients worldwide are commonly receiving considerably less than current protein recommendations, especially during early ICU stay [4, 14]. Accordingly, experts conclude that enteral feeds with a higher protein-to-energy ratio should be chosen for critically ill patients [4, 6].

In the present prospective feasibility tolerability study, we investigated the use of a new enteral formula with a high protein-to-energy ratio (HP:E formula), providing 82 g protein per 1000 kcal consisting of 100% whey protein hydrolysate, developed to meet the increased protein requirements of critically ill patients while avoiding energy overfeeding. The study was primarily aimed to assess the tolerability of the new formula in ICU patients and, secondly, to investigate whether patients receiving the HP:E enteral formula achieved their protein targets faster compared to a matched conventional therapy group (CTG) receiving an established standard enteral formula in our ICU with lower protein content. The aim was to achieve individualized protein targets of ≥1.3 g/kgBW/d with the HP:E formula within 96 h after the initiation of feeding.

## Methods

### Design, setting, and participants

Critically ill patients admitted to the Surgical Intensive Care Unit of the University Hospital of Basel, Switzerland, between May 2016 and January 2017 were enrolled in this prospective, observational tolerability study. Patients aged 18 years and older were included if they were expected to stay at least 96 h in the ICU and were in need of EN during this period. Patients were excluded from the study for any of the following reasons: any oral intake, BMI ≤ 18 kg/m² or ≥35 kg/m², mechanical intestinal obstruction, intestinal perforation, severely impaired liver function, terminal state of cancer, ≥0.5 µg/kg/min norepinephrine upon admission, pregnancy or contraindication for protein-rich nutrition (e.g., severe liver dysfunction). Although the study was carried out in a surgical ICU, medical patients fulfilling the inclusion criteria were eligible for enrollment too.

Informed consent was obtained from an independent physician and from the patient or his next of kin. This study has been approved by the Ethics Committee of Northwestern and Central Switzerland and was conducted in compliance with the protocol, the current version of the Declaration of Helsinki, the ICH-GCP, or ISO EN 14155 (as far as applicable) as well as all national legal and regulatory requirements.

### Interventions

Patients prospectively enrolled in the high protein group received EN with the HP:E formula (Fresubin® Intensive, Fresenius Kabi, Bad Homburg, Germany), providing 1220 kcal and 100 g whey protein hydrolysate per 1000 ml, for the duration of four study days or less in case of adequate oral food intake, discharge or death. The HP:E formula contains 24 energy-percent, fat, thereof ~40% as medium-chain triglycerides (MCT) and 42 energy-percent slow-release carbohydrate blend (isomaltulose and maltodextrin). The nutritional composition of the HP:E formula is shown in Table 1. The study group was matched retrospectively with an equal number of patients who were admitted to the ICU in the same period of time and receiving enteral nutrition for over four days with a formula containing the same amount of energy but only a standard amount of protein (Isosource Protein Fiber Neutral Smartflex®, Nestlé, Vevey, Switzerland) providing 1200 kcal and 67 g protein per 1000 ml. In order to ensure the CTG’s comparability, we matched for APACHE (Acute Physiology And Chronic Health) II score and gender. None of the patients in either group received PN.

**Table 1:** Nutritional composition of the HP:E formula (amounts given per 100 ml).

Energy	122 kcal
Carbohydrates	42%
Protein	33%
Fat	24%
Fibers	1%
Carbohydrates	12.9 g
of which sugars	8.0 g
of which lactose	≤0.5 g
Protein	10.0 g
of which whey protein hydrolysate	10.0 g
Nitrogen	1.57 g
Fat	3.2 g
MCT	1.28 g
LCT	1.92 g
Saturated	0.38 g
Monounsaturated	0.82 g
Polyunsaturated	0.72 g
Fibers	0.64 g
Water	80.5 ml
Osmolarity	600 mosmol/l
Osmolality	740 mosmol/kg H_2_O
Minerals	
Sodium	175 mg/7.6 mmol
Potassium	295 mg/7.5 mmol
Chloride	160 mg/4.5 mmol
Calcium	105 mg/2.6 mmol
Magnesium	30 mg/1.2 mmol
Phosphorus	70 mg/2.3 mmol

All patients included in the study received the EN via nasogastric tube by continuous infusion over 24 h with an hour interval every 4–6 h to check for gastric residual volume (GRV). According to the local ICU feeding protocol [26], EN was initiated in all patients within 24–48 h after admission. After hemodynamic stabilization and under the precondition of a functioning gut, EN was started at an initial feeding rate/volume of 20 ml/h gradually increased by 10 ml/h every 4–6 h until the calculated target volume was reached. Current ESPEN guidelines recommend that gastrointestinal feeding should be delayed when GRV > 500 ml/6 h [1]. In order to anticipate possible intolerance, upon definition of study design authors chose a more conservative approach and decided not to increase the feeding rate with GRV ≥ 300 ml and to prescribe 10 mg of metoclopramide IV three times daily. In case this treatment failed to enhance gastric motility the patient additionally received erythromycin 100 mg IV four times a day. Ventilated patients did not receive any neuromuscular blocking agents.

The nutritional target for the HPG was to achieve a protein intake of ≥1.3 g/kgBW/d within 96 h after the initiation of feeding and to compare achievements to the CTG.

For the calculation of target volumes, the patient’s BW was adjusted according to the body mass index (BMI). For patients with BMI values between 18.1 kg/m² and 19.9 kg/m², a BW corresponding to a BMI of 22 kg/m² was used for all calculations. For patients with a BMI ≥ 30 kg/m² to 34.9 kg/m², the calculations were made with a BW corresponding to a BMI of 25 kg/m². For all other patients, we used the actual BW. Since IC was not available at our institution, an approximate energy target according to recommendations [2, 16] was used.

### Data collection

Upon inclusion, the following characteristics were recorded: admission category (medical or surgical) and diagnosis, relevant co-existing diseases prior to admission (i.e., diabetes, kidney disease, bedsores), as well as GRV and duration of mechanical ventilation. Patients in the HPG were further monitored for diarrhea, emesis, and laxatives/stool softeners in order not to miss any kind of intolerance. Laboratory values were measured at least once per day for 96 h after the feed initiation.

Laboratory values to calculate the APACHE II score, the SAPS II (Simplified Acute Physiology score) score, and the NUTRIC (nutrition risk in critically ill) score were recorded during the first 24 h of ICU stay. Laboratory values for calculation of the sequential organ failure assessment (SOFA) score were recorded on a daily basis. Vital signs were monitored and automatically transmitted to the patient data management system and surveyed by a study investigator. Weight prior to admission was either obtained from the patient, relatives, general practitioners, and height was measured. The patient’s BMI was calculated in order to determine the individual nutritional targets. Study data were recorded until 96 h after the initiation of EN.

NUTRIC score was calculated with the omission of IL-6 levels as a validated tool for the identification of critically ill patients most likely benefiting from artificial nutrition [27].

The intensive care nursing staff recorded data on protein and energy intake from EN using an electronic patient data monitoring system (MetaVision®, Version V, iMDsoft; Tel Aviv, Israel).

### Outcomes

The primary outcome of our study was the GI tolerability of the HP:E enteral formula during the first 4 days of continuous enteral feeding. Intolerance was defined as ≥300 ml GRV per day, vomiting or the occurrence of diarrhea, defined as ≥3 loose or liquid stools per day during the first 2 days. Diarrhea was only considered as a sign of GI intolerance during the first 48 h after initiation of EN. Patients without any bowel movements were prescribed laxatives by the third day according to local practice and therefore were prone to develop loose stools, which could not be distinguished from diarrhea related to the intake of the study formula.

Secondary outcomes were the total amount of protein and energy delivered over the first 96 h of feeding as well as the percentage of patients reaching their protein target in the HPG compared to the CTG.

According to the treatment protocol of the surgical ICU, physiotherapists started moving patients’ limbs passively for 30 min from day two after admission in case their condition allowed it.

### Data analysis and statistics

Descriptive data are reported as absolute values, mean ± standard deviation (SD) if appropriate, or median and interquartile range (IQR) or percentage.

We estimated a sample size including 10% loss to follow-up of 25 patients in total for a power of 0.8 to see a modest difference in protein or energy uptake in groups with a significance level of alpha of 0.05.

Data analysis on differences in protein and energy intake was performed with two-way mixed ANOVA for time, group and interaction. Data met the conditions of normality and homoscedasticity as assured by visual inspection using QQ-plots and Levene’s test. Upon significant ANOVA findings, post-hoc *t*-tests for differences of group means were performed with a Bonferroni correction.

Treating doctors and nursing staff were not part of the study team and therefore independent in their medical decisions.

## Results

### Enrollment and baseline characteristics

**Fig. 1:**
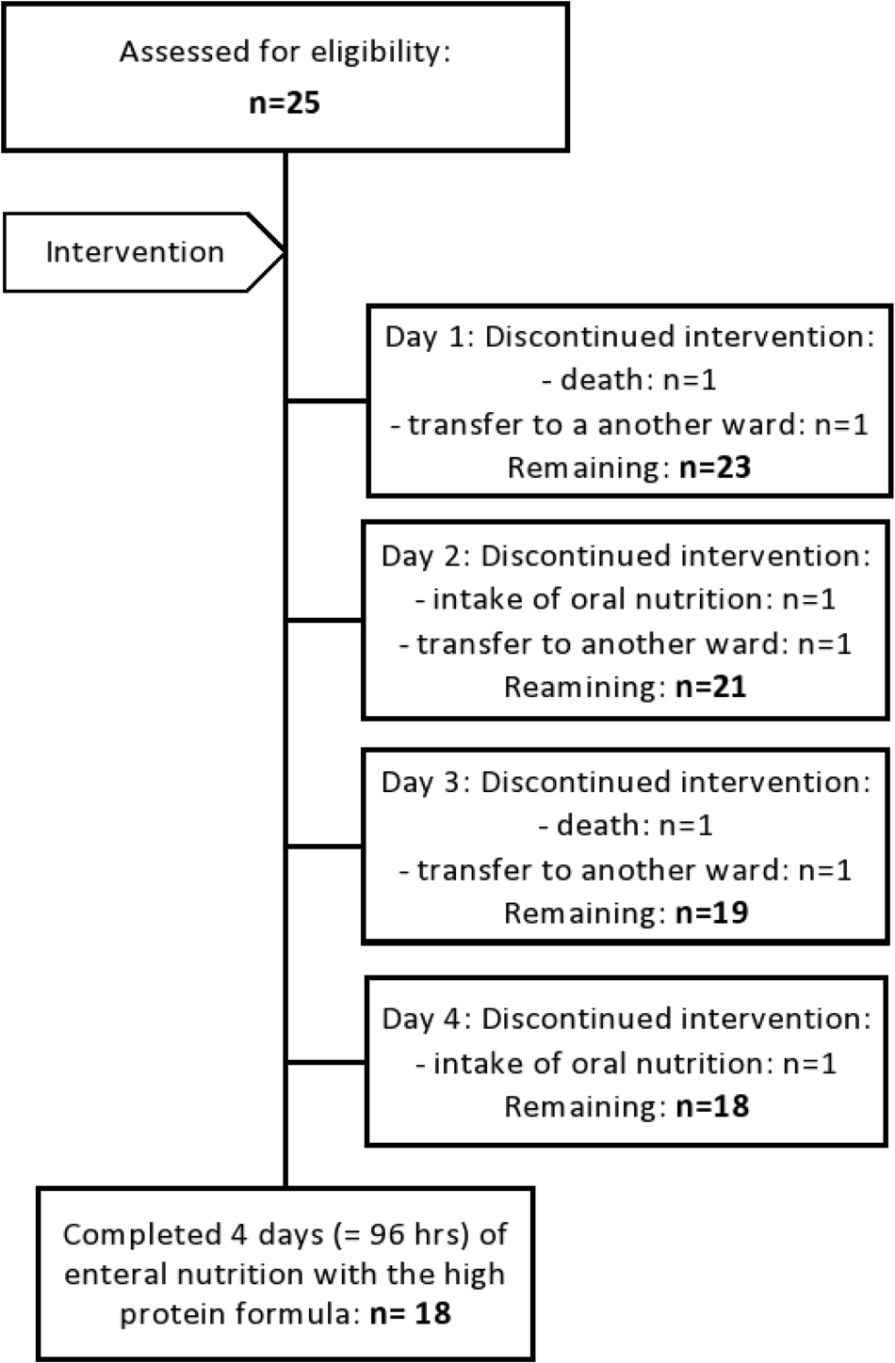
Consort diagram of included study patients showing the patient flow through the HPG. HPG, high protein group; hrs, hours.

Twenty-five patients were enrolled in the HPG to receive the HP:E enteral formula. Two patients had to be excluded on the first study day: one patient died and one patient achieved adequate oral food intake and was transferred to a regular ward. Over the intervention period, two more patients dropped out by day 3 and three more patients by day 4, due to death (*n* = 1) or transfer to a regular ward with adequate oral intake (*n* = 4). Hence, the final intervention group consisted of 18 patients eventually who completed 4 days of feeding with the high protein enteral formula. Participant flow through the HPG is shown in Fig. 1. The HPG was matched with 18 patients during the same period of time who received EN with the conventional formula for at least 4 days. We matched the group for APACHE II score and gender on the basis of our eligibility criteria: at baseline, the median APACHE II score of both groups was 21.0 (IQR) for HPG 19.0–24.8, for CTG 19.3–25.0 equal to a mortality risk of 40%. In both groups, there were 4 female and 14 male patients.

Patients in the HPG and CTG (Table 2) were also comparable with regard to baseline demographic characteristics like age, weight and BMI, enteral nutrition risk (NUTRIC score), and organ failure (SOFA score)*.* The most frequent admission category in both groups was post-cardiac surgery.

**Table 2:** Baseline characteristics and admission diagnoses reported as median (IQR) or mean ± SD as *N* (%).

Baseline characteristics *N*	High protein formula group (HPG) 18	Conventional formula group (CFG) 18
Elective/emergency	1/17	5/13
Age, years	73.0 (56.3–76.0)	70.0 (67.0–75.8)
Gender, f/m	4/14	4/14
Weight, kg	78.5 (72.8–93.8)	80.0 (71.3–96.0)
BMI, kg/m^2^	27.8 (23.1–31.8)	26.9 (23.4–31.3)
NUTRIC score	6.0 (4.3–6.0)	5.5 (4.0–6.0)
Low (0–5)	8	9
High (6–10)	10	9
APACHE II	21.0 (19.0–24.8)	21.0 (19.3–25.0)
SOFA score	8.9 ± 2.6	9.1 ± 3.4
Requirement for MV	D1: 16/18 (89%) D4: 15/18 (83%)	D1: 18/18 (100%) D4: 9/18 (50%)
Admission diagnosis		
Post cardiac surgery	7 (39%)	8 (44%)
Trauma	4 (22%)	1 (6%)
Sepsis	2 (11%)	0 (0%)
Respiratory failure	2 (11%)	2 (11%)
Other	3 (17%)	7 (39%)

*MV* mechanical ventilation.

### Tolerance of enteral feeding (primary outcome)

Overall six patients in each group experienced a GRV of ≥300 ml, which corresponds to 33.3% (Table 3). In the HPG, three patients (16.7%) experienced a GRV of ≥500 ml on one of four study days, in the CTG there was one patient (5.6%) affected by a GRV of ≥500 ml. On days 1 and 2, 61.1% and 83.3% of patients respectively, received propulsive medication (metoclopramide, erythromycin) and/or stool softeners (lactulose) in the HPG according to the routine protocol of the surgical ICU and 27.3% were given laxatives on the first two days. Two more patients were suffering from emesis and vomiting lasting for 1–2 days (Table 3). Medication, diarrhea, and vomiting were not recorded in the CTG. There was no need for discontinuation of EN in any patient in either group.

**Table 3:** Incidence of feeding intolerance reported as *N* (%) with the HP:E formula vs. controls receiving a standard enteral formula. Diarrhea defined as ≥3 loose or liquid stools per day.

	HP:E formula (HPG)	Standard formula (CTG)
GRV ≥300 ml (any day)	6 (33.3%)^a^	6 (33.3%)^a^
GRV ≥300 ml (day 1)	3 (16.7%)	–
GRV ≥300 ml (day 2)	2 (11.1%)	–
GRV ≥300 ml (day 3)	1 (5.6 %)	–
GRV ≥300 ml (day 4)	2 (11.1%)	–
GRV ≥500 ml (any day)	3 (16.7%)	1 (5.6%)
Diarrhea (≥1000 ml, any day)	2 (11.1%)	NA
Emesis (number of patients)	2 (8.7%)	NA
EN discontinuation	0	0

^a^Patients having GRV ≥300 ml/d.

*CTG* conventional therapy group, *EN* enteral nutrition, *GRV* gastric residual volume, *HPG* high protein group, *NA* data not available.

### Protein and energy intakes

The average time from admission to initiation of enteral feeding after hemodynamic stabilization was 37 ± 23 h in the HPG and 40 ± 28 h in the control group. In both groups, 14 patients (78%) received EN within 48 h following admission. On average, patients in the HPG received 0.4 g/kgBW/d of protein with the HP:E formula during the first 24 h after admission (Table 4). On the second day, the average protein intake in the HPG increased to 1.1 g/kgBW/d. Within 72 h after initiation of feeding, patients in the HPG achieved their protein target of ≥1.3 g/kgBW/d with an average of 1.5 g/kgBW/d. On days 3 and 4, 72% of patients in the HPG met their individual daily protein target. In contrast, patients in the CTG received an average of 0.9 g of protein/kgBW/d by day 3 and 4 and only 22% and 33%, respectively, reached their individual protein intake target of ≥1.3 g/kgBW/d (Table 5). The HPG met the average caloric target of 20 kcal/kgBW/d on day 3 with 101% and on day 4 with 97%. A total of 61% of patients reached their individual target on both days, whereas in the CTG, 108% and 114% met the average target by day 3 and 4, respectively, and 67% of patients reached their individual target of 20 kcal/kgBW/d (Table 5). On each study day, the average feeding volume was lower in the HPG vs. the CTG (Table 4).

**Table 4:** Energy and protein intakes over the first 96 h of enteral feeding reported per study day, high protein group (HPG) vs. control group (CTG). Values are given as mean intakes over 24 h reported per kgBW, as total daily intakes, and as a percentage of patients reaching their protein/energy targets on each study day.

	HPG		CTG	
Hours	Energy intake [kcal/kg]	CI	Energy intake [kcal/kg]	CI
24	4.9	2.4–5.7	7.3	5.0–9.1
48	14.7	11.1–20.5	18.3	15.8–21.1
72	20.1	17.2–23.0	21.6	18.1–24.6
96	19.4	13.6–23.8	22.8	19.1–27.1
	**Total energy [kcal]**	**CI**	**Total energy [kcal]**	**CI**
24	372.9	215.3–436.8	612	460–764
48	1067.0	812.2–1360.0	1481	1311–1651
72	1499.0	1220.9–1878.8	1714	1587–1840
96	1443.0	882.7–1773.0	1805	1662–1949
	**Percentage of energy target [%]**	**CI**	**Percentage of energy target [%]**	**CI**
24	24	15–37	36	25–45
48	71	52–94	94	79–105
72	105	80–116	106	90–123
96	104	87–109	109	95–136
	**Protein intake [g/kg]**	**CI**	**Protein intake [g/kg]**	**CI**
24	0.4	0.2–0.5	0.3	0.2–0.4
48	1.1	0.8–1.6	0.7	0.4–1.0
72	1.5	1.2–1.8	0.9	0.7–1.2
96	1.5	1.0–1.8	0.9	0.7–1.2
	**The total amount of protein [g]**	**CI**	**The total amount of protein [g]**	**CI**
24	30.6	17.7–35.8	29.6	22.5–35.5
48	87.5	66.6–111.5	74.9	63.4–83.6
72	122.9	100.1–154.0	88.1	76.4–99.3
96	118.3	72.4–145.3	93.0	83.9–105.5
	**Percentage of protein target [%]**	**CI**	**Percentage of protein target [%]**	**CI**
24	23	14–37	28	19–35
48	71	52–94	73	61–82
72	105	80–116	82	70–95
96	104	87–109	85	0.74–1.05
	**Feeding Volume [ml]**	**CI**	**Feeding Volume [ml]**	**CI**
24	307	177–358	414	335–530
48	921	666–1115	1089	946–1248
72	1294	1001–1540	1325	1140–1482
96	1257	724–1453	1352	1253–1574

**Table 5:** Percentage of patients reaching their energy and protein goals after 24, 48, 72, and 95 h of enteral feeding, respectively (calculations are rounded to one digit after the decimal point for better readability).

	HPG (*n* = 18)		CTG (*n* = 18)	
	Patients reaching ≥20 kcal/kg/day energy (%)	Patients reaching ≥1.3 g/kg/day protein (%)	Patients reaching ≥20 kcal/kg/day energy (%)	Patients reaching ≥1.3 g/kg/day protein (%)
24 h	0	0	0	0
48 h	27.8	38.9	33.3	5.6
72 h	61.1	72.2	66.7	22.2
96 h	61.1	72.2	66.7	33.3

**Fig. 2:**
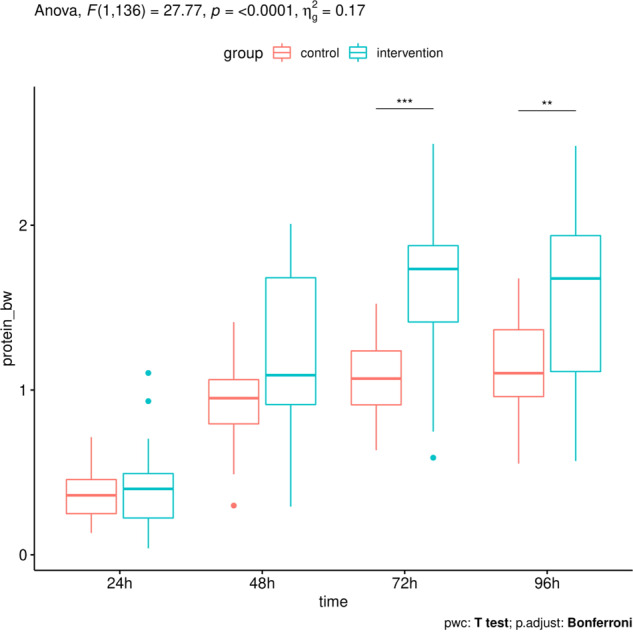
Boxplots of for protein intake per kgBW over 24, 48, 72, and 96 h using two-way ANOVA with Bonferroni correction. ANOVA, analysis of variance; BW, body weight.

**Fig. 3:**
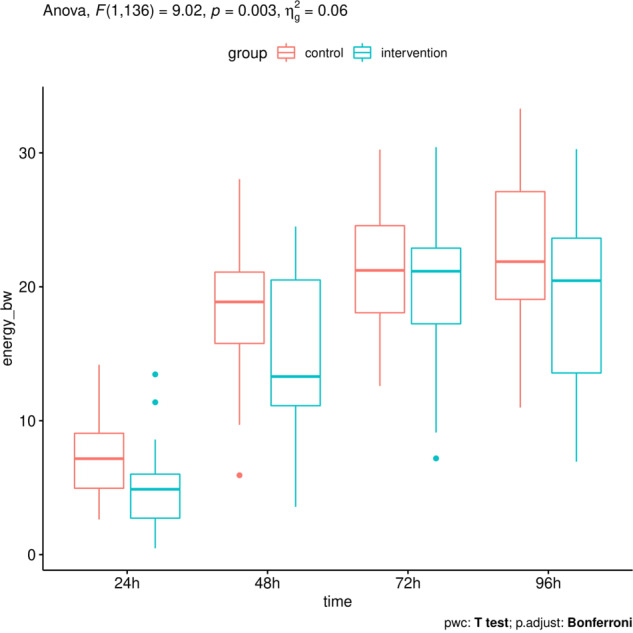
Boxplots for energy intake per kgBW over 24, 48, 72, and 96 h using two-way ANOVA with Bonferroni correction. ANOVA, analysis of variance, BW, body weight.

Analysis of variance respecting repeated measures at different time points within groups showed a significant main effect of time with an *η*_*g*_^2^ > 0.5, i.e., explaining more than 50% of the variance, for all measurements. The main effect of the group was also significant for all measurements, explaining up to 17% of the variance (e.g., BW adjusted protein intake *F* = 27.77, *p* ≤ 0.0001, *η*_*g*_^2^ = 0.17). HPG thus showed higher amounts of protein (mean = 4.5 g) compared to the CTG (mean = 2.8 g). The interaction term was not significant in any analysis. Considering the post-hoc testing, it can be seen that group differences in protein intake between HPG and CTG were significant at *t* = 72 h and *t* = 96 h (Fig. 2). As expected, there were not any group differences in energy intake (Fig. 3).

### Glycaemic control and laboratory values

The median peak daily glucose level in the HPG was 8.9 mmol/l (8.6–11.1 mmol/l) with glucose being the highest on day 4 with 10.1 mmol/l. In the CTG, the median peak daily glucose level was 10.3 mmol/l (9.2–11.2 mmol/l), the highest value was scored on day 2 (Table 6). None of the patients needed more than 25 units of insulin per day, regardless of the presence of diabetes prior to admission. Table 6 shows the course of selected laboratory values over time in the HPG. Plasma ammonium was slightly elevated, yet remained within normal range, at 72 and 96 h after initiation of enteral feeding. Triglycerides remained stable and within normal range throughout the study period. The median plasma creatinine level was a little over the upper limit of the normal range (42–80 µmol/l) and ranged from 104 to 125 mcmol/l over the 4 days with a median of 116 mcmol/l on day 4. In the CTG, creatinine remained in the normal range throughout the study. Urea increased in both groups over the study period and values were above the upper limit of a normal range (3.0–7.8 mmol/l) throughout the study in the HPG and from 72 h after initiation of enteral feeding until the end of the study period in the CTG.

**Table 6:** The course of specific laboratory values over time in the HP:E formula (HPG) and control formula (CTG) groups reported as median (IQR). Values in brackets indicate the normal range.

	Admission	24 h	48 h	72 h	96 h
Peak daily blood glucose (6–10 mmol/l)					
HPG	9.1 (7.2–11.4)	9.1 (7.7–10.3)	9.1 (7.7–9.6)	9.2 (7.9–10.5)	10.1 (9.0–10.6)
CTG	NA	8.8 (7.4–10.3)	11.2 (9.1–12.4)	9.9 (9.0–12.5)	9.7 (8.9–10.8)
Ammonium (15–55 μmol/l)					
HPG	33 (27–39)	34 (29–39)	32 (25–49)	43 (25–49)	39 (34–48)
CTG	NA	NA	NA	NA	NA
Creatinine (50–110 μmol/l)					
HPG	111 (90–152)	104 (89–154)	119 (95–152)	125 (96–166)	116 (82–160)
CTG	75 (55–88)	77 (62–88)	69 (53–86)	64 (47–91)	65 (47–89)
Triglycerides (<1.8 mmol/l)					
HPG	1.3 (1.02–1.53)	1.3 (1.03–1)	1.2 (1.25–2.16)	1.4 (1.03–2.28)	1.4 (0.88–1.6)
CtG	NA	NA	NA	NA	NA
Urea (2.5–7.1 mmol/l)					
HPG	9.6 (6.0–12.1)	10.0 (5.9–12.5)	11.4 (7.1–17.4)	12.1 (7.9–17.0)	14.2 (8.3–18.1)
CTG	5.8 (4.5–8.6)	6.1 (5.2–9.0)	7.4 (6.2–8.0)	8.4 (6.3–9.5)	9.0 (6.0–10.8)
24 h UUN (83–532 mmol/24 h)					
HPG	NA	107 (53–163)	174 (148–270)	NA	NA
CTG	NA	NA	NA	NA	NA

*UUN* urinary urea nitrogen, *NA* not available.

### Nitrogen excretion

Twenty-four-hour urinary urea nitrogen (UUN) excretion was assessed in the HPG after 24 and 48 h feeding time, respectively, and was well within the normal range (Table 6). Setting the UUN excretion rates at 24 and 48 h against the enteral protein intakes over the respective time intervals (31 and 88 g, corresponding to 5.0 and 4.1 g nitrogen, respectively), the calculated nitrogen balance was −1.3 g/d after 24 h and 5.5 g/d after 48 h according to the formula by Blackburn and Bistrian [28].

## Discussion

The findings of the BEntHiPro prospective tolerability study revealed that the new HP:E enteral formula with 100% whey protein hydrolysate appears to be equally well tolerated in terms of GRV in critically ill patients compared to an established enteral formula. In addition, a protein target of ≥1.3 g/kg BW/d could be reached within 72 h after initiation of enteral feeding in 72% of patients with the administration of this enteral formula, while targeted energy intake was reached in 61%.

As would be expected, we found a significant effect of time for both energy and protein intake with repeated measures showing consistent time courses for each subject. The effect of the group was also found to be significant in protein intake corrected for BW. The interaction between time and group was not significant, which was to be expected for the total patient number and suspected modest effect. In addition, more subtle differences in protein intake at earlier time points may have been identified with more power, i.e., a higher total number of patients.

In terms of overall GI intolerance including emesis and diarrhea, patients did not experience gastrointestinal intolerance requiring interruption of enteral nutrition in any patient. However, as it turned out during our tolerability study, diarrhea did not qualify as the primary outcome for tolerability since 83% of all patients received several laxatives and/or stool softeners which considerably biased the interpretation of diarrhea as an “adverse event”. The incidence of GI intolerance in terms of high GRV with the HP:E formula was slightly lower compared to a previous study with the same product, reporting high GRVs (>250 ml) in 15% of ventilated ICU patients over the first 4 days [29]. In contrast, a recent RCT investigating an intact protein enteral formula providing 63 g of mixed intact protein from whey, casein, pea, and soy per 1000 ml over 10 days in a comparable patient setting reported a considerable greater incidence of GI intolerance with high GRVs (>500 ml) in 18.2% of patients [30].

Rice et al. [31] also conducted a study with an increased amount of protein in their randomized open-label trial using an experimental formula with high-whey peptides (37%) and low carbohydrates (29%) compared to a standard formula with 25% of protein and 45% of carbohydrates. On days 1–5, they provided 1.1 ± 0.3 g/kg/ IBW/d and 1.2 ± 0.4 g/kg IBW/d protein, respectively. Although they reported an increase in the number of patients showing abdominal distension in the high-protein group, they did not record a difference in the number of patients requiring EN discontinuation.

With regard to the secondary study outcomes (i.e., total amounts of protein and energy delivered over the first 96 h after initiation of enteral feeding), the HP:E formula proved to be clearly superior to an established enteral formula in terms of providing adequate amounts of protein according to individual targets in line with current guidelines and expert recommendations for critically ill patients [1, 2, 13, 14]: 67% of patients reached protein targets ≥1.3 g/kgBW/d within 4 days after initiation of enteral feeding. Importantly, protein targets were achieved in the HPG while the provision of energy did not overly exceed requirements (not more than 101% of the goal amount).

In the present investigation, more than half of all patients (56%) in the HPG should be expected to benefit particularly from early high protein nutrition since their NUTRIC score indicated a high nutrition risk.

Whey protein is rated as a “fast protein” producing a rapid and pronounced postprandial rise in blood amino acids, in particular of leucine [32–34]. In the study by Looijaard et al. [29], significant increases in plasma levels of essential amino acids, including branched-chain amino acids and leucine, were seen on study days 2 and 4 vs. baseline. Thus, the 100% whey protein hydrolysate-based enteral formula effectively increased the availability of essential amino acids for protein synthesis during the early phase of critical illness. Tracer studies have previously confirmed that protein derived from early enteral nutrition is effectively used in critically ill patients for body protein accretion during the first week of ICU treatment [35]. Nitrogen balances calculated from UUN excretion rates in the present study suggest that a state of nitrogen anabolism was reached with the HP:E formula already within the first 48 h. This finding indicates that the amino acids derived from the whey protein hydrolysate administered were largely retained in the body to counteract protein catabolism.

In contrast, other protein sources are less suitable to meet the specific needs of critically ill patients. Casein is prone to coagulation, delaying gastric emptying and absorption [36]. Pea protein is considerably lower than whey protein in branched-chain amino acids and in leucine while being deficient in sulfur-containing amino acids [37]. In the study by van Zanten et al. [30], investigating an enteral formula containing a mixture of intact proteins derived from whey, casein, pea, and soy, a higher increase in total serum amino acids was reported on day 5 with the test product compared to a standard high protein formula. Yet, this study did not report any data on specific amino acids, in particular on essential amino acids and leucine.

Ammonium and triglyceride parameters did not show any clinically relevant changes or abnormalities in response to four days of feeding with the HP:E enteral formula. There was no hyperammonaemia throughout the study, indicating that, in this group of critically ill patients, the amounts of nitrogen administered over the first 96 h of feeding did not exceed the functional capacities of the liver and kidneys. BUN increased over the study period in both groups and, in the HPG, values were above the normal range already at baseline, as it is frequent in critical illness [38, 39]. In the present study, there were five patients with AKI grade I, defined as creatinine ≥1.5 times of normal, at 96 h in the HPG. No patient developed AKI in the CTG. This finding needs careful monitoring in a subsequent larger trial in order to find out whether this difference in acute kidney injury is due to the small size of this pre-trial, due to a selection bias, or due to the higher amount of protein administered. In relation to this, it should also be examined whether the increased urea synthesis in the HPG is due to the enhanced amino acid oxidation, which has been observed in critical illness [40]. However, in a recently published study comparing high-protein and non-high-protein nutrition in critically ill patients, Suzuki and colleagues concluded that the high-protein group had significantly lower 28- and 90-day mortality and significantly greater delta-BUN, also after propensity score matching, but that delta-BUN might not be associated with outcomes [41].

We acknowledge that the study has several limitations. First, because the study was conceived as a tolerability study, only a small number of patients were included and as more patients dropped out during the intervention than expected, the study was not powered to assess any effects of the study nutrition on major clinical outcomes. Larger randomized trials are needed to confirm its tolerance and effect on patient outcomes. Second, this was not an RCT, but findings with HP:E formula were compared to a matched CTG with similar disease characteristics and feeding targets receiving an established enteral formula. Third, the meaningfulness of the findings is also limited by the fact that information on patients’ BW, required for all calculations of energy and protein targets, was obtained from questioning the patient, relatives, or general practitioners for reasons of practicability in an ICU setting. Also, patients at the obese end of the BMI spectrum were excluded. According to the guidelines, patients with BMI > 40 kg/m² require 11–14 kcal and up to 2.5 g protein/kg IBW [2]. For a patient with a BMI of 40 kg/m² (170 cm, 120 kg) this would translate to 1320–1680 kcal and 180 g protein. The high-protein product provides 100 g of protein in 1220 kcal. Therefore, we would not reach the protein target with this product.

As a matter of fact, the amount of protein allocated should be derived by the lean body mass rather than the actual BW as it is still customary for most studies including this observational tolerability study and the studies mentioned [42]. It is about the protein pool of the body that we want to stop from draining which differs substantially from the whole body mass. It has been known for some decades now that total body skeletal muscle mass can be accurately measured by the third lumbar vertebra (L3) on abdominal computed tomography (CT) [43, 44]. Virtually any patient hospitalized in the ICU has a CT of the abdomen and it is unclear why this measurement has not been used more widely. Finally, findings with regard to nitrogen balance must be interpreted with caution since the classic nitrogen balance estimation [28] underestimates non-urea urinary nitrogen losses in many critically ill patients [45]. Moreover, the suitability of nitrogen balances as a measure of protein adequacy in critically ill patients has been questioned due to the associated risk of nitrogen intake over- and nitrogen excretion underestimation and the difficulty in obtaining a steady state over short periods of time (48 h) [14, 46–48]. Nevertheless, as several authors have pointed out correctly [22, 49, 50]: not every protein-ratio fits every critically ill patient. The loss of protein depends on the disease itself, age, and weight, to name but a few.

With the above-demonstrated insights made with this prospective observational study, consent has already been obtained from the responsible Ethics Committee to perform the double-blind, randomized controlled clinical trial to compare an HP enteral formula with a standardized high carbohydrate product on total calorie and protein intake which will include the patients recruited for this trial.

## Conclusion

An enteral formula with an HP:E ratio containing 33% whey protein hydrolysate as a protein source appears to be suitable for early enteral feeding of critically ill patients requiring mechanical ventilation. However, larger and randomized trials are needed in order to recommend its use as a standard of care in the ICU. Due to the good GI tolerance, individual protein targets in line with current recommendations (≥1.3 g/kgBW/d) could be met within 72 h after initiation of enteral feeding with the HP:E formula, while, over the same period of time, energy provision was virtually met, but did not exceed a moderate caloric target of 20 kcal/kgBW/d. The new HP:E formula is thus in line with recent expert statements calling for enteral feeds with a higher protein-to-energy ratio to meet current guidelines for critical illness while the risk of energy overfeeding and hyperglycemia is minimized.

## Data Availability

Prof Martin Siegemund had full access to all data in the study and takes responsibility for the integrity of the data and the accuracy of the data analysis.
